# Identified five variants in CFTR gene that alter RNA splicing by minigene assay

**DOI:** 10.3389/fgene.2025.1543623

**Published:** 2025-03-20

**Authors:** Bingying Zhang, Yiyin Zhang, Yan Zhang, Xuyan Liu, Ran Zhang, Zhi Wang, Fengjiao Pan, Ning Xu, Leping Shao

**Affiliations:** ^1^ School of Clinical Medicine, Shandong Second Medical University, Weifang, China; ^2^ Department of Nephrology, The Affiliated Qingdao Municipal Hospital of Qingdao University, Qingdao, China; ^3^ Department of Nephrology, Peking University Medical Lu Zhong Hospital, Zibo, China; ^4^ Department of Nephrology, Qingdao Eighth People’s Hospital, Qingdao, China

**Keywords:** CFTR, minigene assay, pre-mRNA splicing, exonic variant, exon skipping

## Abstract

**Background:**

Cystic fibrosis (CF) is a common monogenic multisystem disease caused primarily by variants in the CFTR gene. Emerging evidence suggests that some variants, which are described as missense, synonymous or nonsense variants in the literature or databases, may be deleterious by affecting the pre-mRNA splicing process.

**Methods:**

We analyzed 27 exonic variants in the CFTR gene utilizing bioinformatics tools and identified candidate variants that could lead to splicing changes through minigene assays. Ultimately, we selected eight candidate variants to assess their effects on pre-mRNA splicing. The numbering of DNA variants is based on the complementary DNA (cDNA)sequence of CFTR (Ref Seq NM_000492.4).

**Results:**

This study assessed the impact of CFTR variants on exon splicing by combining predictive bioinformatics tools with minigene assays. Among the eight candidate single nucleotide alterations, five variants (c.488A>T,c.1117G>T, c.1209G>T, c.3239A>G and c.3367G>C) were identified as causing exon skipping.

**Conclusion:**

Our study employed a minigene system, which offers great flexibility for assessing aberrant splicing patterns when patient mRNA samples are not accessible, to investigate the effects of exonic variants on pre-mRNA splicing. Our experimental outcomes highlight the importance of analyzing exonic variations at the mRNA level.

## 1 Introduction

Cystic fibrosis (CF) is an autosomal recessive disorder resulting from variants in the cystic fibrosis transmembrane conductance regulator (CFTR) gene, which encodes a cAMP-regulated chloride channel protein. The clinical manifestations of CF include pulmonary disease, pancreatic insufficiency, male infertility, and elevated chloride levels in sweat. The severity of the disease is highly variable, with some patients exhibiting only a subset of the classic CF symptoms. The CFTR gene comprises 27 exons spanning approximately 250 kb of DNA located on chromosome 7q31.2. The CFTR protein consists of two transmembrane domains (TMDs) that form the ion channel, two cytoplasmic nucleotide-binding domains (NBDs) responsible for binding and hydrolyzing adenosine 5′-triphosphate (ATP), and a regulatory (R) domain that must be phosphorylated for the channel to open (Liu et al., 2019; Csanády et al., 2019). Over 4,000 CFTR variations have been identified in the latest research (Ideozu et al., 2024). However, these represent only a small fraction of the potential variations in the CFTR gene. With the introduction of Next-Generation Sequencing (NGS) in clinical laboratories for routine diagnosis, the number of identified variants is expected to increase significantly, necessitating careful classification of these variants as either disease-causing or benign. While several variants have been conclusively linked to CF, many remain uncharacterized and are categorized as variants of unknown significance (VUS) (Martin et al., 2021).

RNA splicing is a critical process in the post-transcriptional regulation of gene expression in eukaryotes, wherein the newly synthesized precursor messenger RNA (pre-mRNA) transcript is converted into mature messenger RNA (mRNA). This process entails the removal of introns, executed by the spliceosome-a large, dynamic ribonucleoprotein complex composed of five small nuclear ribonucleoprotein particles (snRNPs: U1, U2, U4, U5, and U6). These snRNPs primarily recognize abundant splicing signals and facilitate the splicing reaction (Warf and Berglund, 2010; Chen and Manley, 2009). Furthermore, exons harbor various splicing regulatory elements, including exonic splicing enhancers (ESEs) and exonic splicing silencers (ESSs), which modulate the identification of splice sites (Cartegni et al., 2002). Beyond the well-documented mechanism by which intron variants directly alter splicing sites (5′ donor and 3′ acceptor sites, as well as branch sites), emerging studies indicate that variants within exons can also disrupt pre-mRNA splicing. These variants can activate novel cryptic splice sites by influencing a range of splicing regulatory signals (Gonzalez-Paredes et al., 2014). Consequently, minigene experiments should be undertaken to validate the splicing effects of variants in CFTR exons.

## 2 Materials and methods

### 2.1 Variant nomenclature

DNA variant numbering is based on the cDNA sequence for CFTR (Ref Seq NM_000492.4). The nomenclature of variants followed the guidelines of the Human Genome Variation Society (http://varnomen.hgvs.org), with c.1 representing the first position of the translation initiation codon.

### 2.2 Bioinformatics analyses and screening criteria

We collected all missense and nonsense variants of the CFTR gene from the Human Gene Mutation Database and ClinVar (as of July 2023). Each variant was analyzed for its potential effects on pre-mRNA splicing using online bioinformatics tools. BDGP (http://www.fruitfly.org/) and Splice AI (https://spliceailookup.broadinstitute.org/) were employed to assess the potential impact of variants on consensus 5′ss splice sites (5′ss), 3′ss splice sites (3′ss), or the activation of novel splice sites. The Human Splicing Finder (HSF; https://www.genomnis.com/access-hsf) was utilized to evaluate the possible effects of putative variants on splicing regulatory elements, such as ESEs and ESSs.

In this study, the screening criteria for single nucleotide substitutions in the CFTR gene were as follows. First, exons with a BDGP score below 0.7 were selected for further analysis. Then, bioinformatics software (BDGP, Splice AI and HSF 3.1) was employed to assess the effects of all single nucleotide variants (SNVs) in these exons on exon splicing sites and splicing regulatory elements, with HSF scores less than −8 considered significant. Besides, potential splicing variants within three bases of the 5′or 3′end of the exon were included (Xin et al., 2022).

### 2.3 Minigene constructions

**FIGURE 1:**
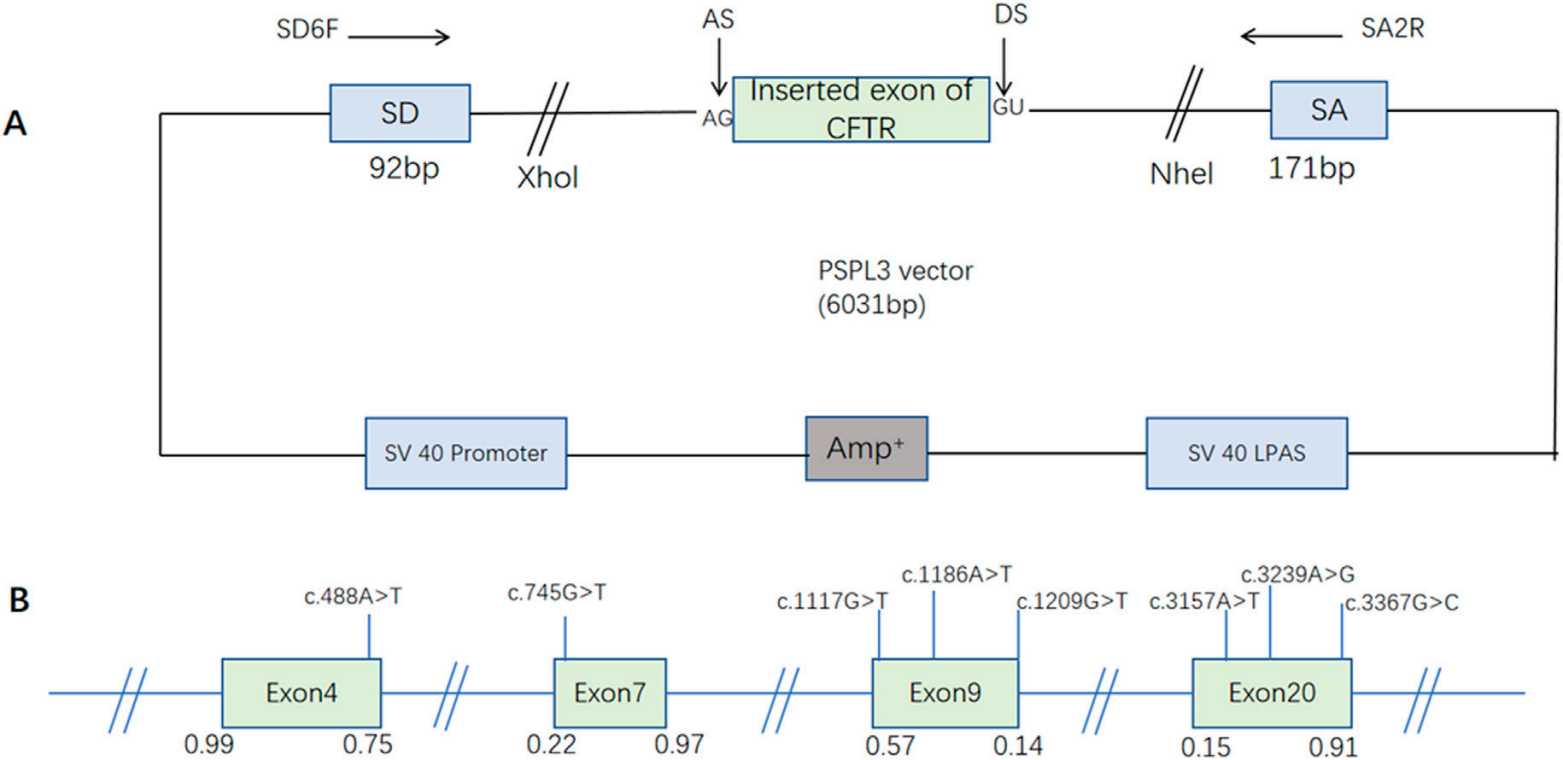
The schematic diagram of the minigene based on the pSPL3 exon trapping vector and the position of eight presumed exonic variants. **(A)** The wild-type and mutant fragments of the target exon were connected to the pSPL3 vector via XhoI and NheI cloning sites of the pSPL3 vector, respectively, to form wild-type and mutant pSPL3 plasmids. **(B)** The position of candidate variants in the CFTR gene. Green boxes and blue lines between them represent the coding exons sequences, respectively. Their sizes are not proportional. The BDGP scores of donor and acceptor splice sites are represented in decimal.

In order to investigate the effect of the candidate single nucleotide alteration on the splicing process, a minigene splicing assay based on the pSPL3 exon capture vector was used for *in vitro* analysis, and minigene constructions have been described as previously reported (Figure 1) (Zhang et al., 2021) Specific primers linking the XhoI and NheI restriction enzyme sites (XhoI: TGGAGCTCGAG; NheI: AATTTGCTAGC) were used to amplify the exons in which the screened variant resides and 50–200 bp of their intronic flanking regions. Then they were cloned into the splicing vector pSPL3 to form WT plasmid. Specific primers for each fragment were designed using Primer X5 (Supplementary Table S1). Selected substitutions were introduced into WT plasmid by site-directed mutagenesis using GeneArt Site-Directed Mutagenesis PLUS System (Thermo Fisher Scientific) as instructed by the manufacturer and mutagenesis primers are listed in Supplementary Table S2. All constructed vectors were transformed into *Escherichia coli* DH5α-competent cells (TaKaRa) for amplification. All constructs were confirmed through Sanger sequencing (as shown in Supplementary Figure S1).

### 2.4 Minigene transfection

HEK 293T and BEAS-2B cells both were purchased from the American Type Culture Collection (ATCC, USA). Both cell types were cultured in DMEM medium (Procell, China) supplemented with 10% fetal bovine serum (Procell, China), penicillin (100 U/L, Procell), and streptomycin (100 mg/L, Procell). The cell cultures were incubated in a humidified incubator at 37°C with 5% CO2. One day prior to transfection, the cells were transferred to 12-well plates and allowed to reach about 70%–80% confluency in a medium devoid of antibiotics. According to the manufacturers’ instructions transfections with empty pSPL3 control, wild-type, and mutant minigenes were carried out using Lipofectamine 2000 (Invitrogen, United States).

After forty-eight hours, total RNA was extracted with RNA-easy Isolation Reagent (Vazyme Biotech Co, Ltd, China). First-strand cDNA was synthesized from 1 *μg* of total RNA by RT-PCR (reverse transcription PCR) using PrimeScript 1st Strand cDNA Synthesis kit (Takara, Japan) under the instruction booklet of manufacturer. The resulting cDNA was amplified by PCR using vector-specific primers: SD6 (the forward primer: 5′-TCT​GAG​TCA​CCT​GGA​CAA​CC-3′) and SA2 (the reverse primer: 5′-ATC​TCA​GTG​GTA​TTT​GTG​AGC-3′). The PCR products were separated by electrophoresis on a 1.5% agarose gel, and the intensity of each band was quantified using the grayscale values from the software ImageJ. We ensured that each measurement fully encompassed the target band while subtracting a representative background. Additionally, we maintained a consistent frame size for all quantified bands to ensure uniformity in our analysis. The transcripts were analyzed by DNA sequencing (as shown in Supplementary Figure S2). The SnapGene software was used to compare DNA sequences with the reference CFTR sequence published in GenBank. When the splicing pattern differed from that of the WT minigene in both HEK 293T and BEAS-2B cells, variations were considered to result in aberrant splicing, and the stability and reliability of the results were verified by three repeated experiments.

## 3 Results

**FIGURE 2:**
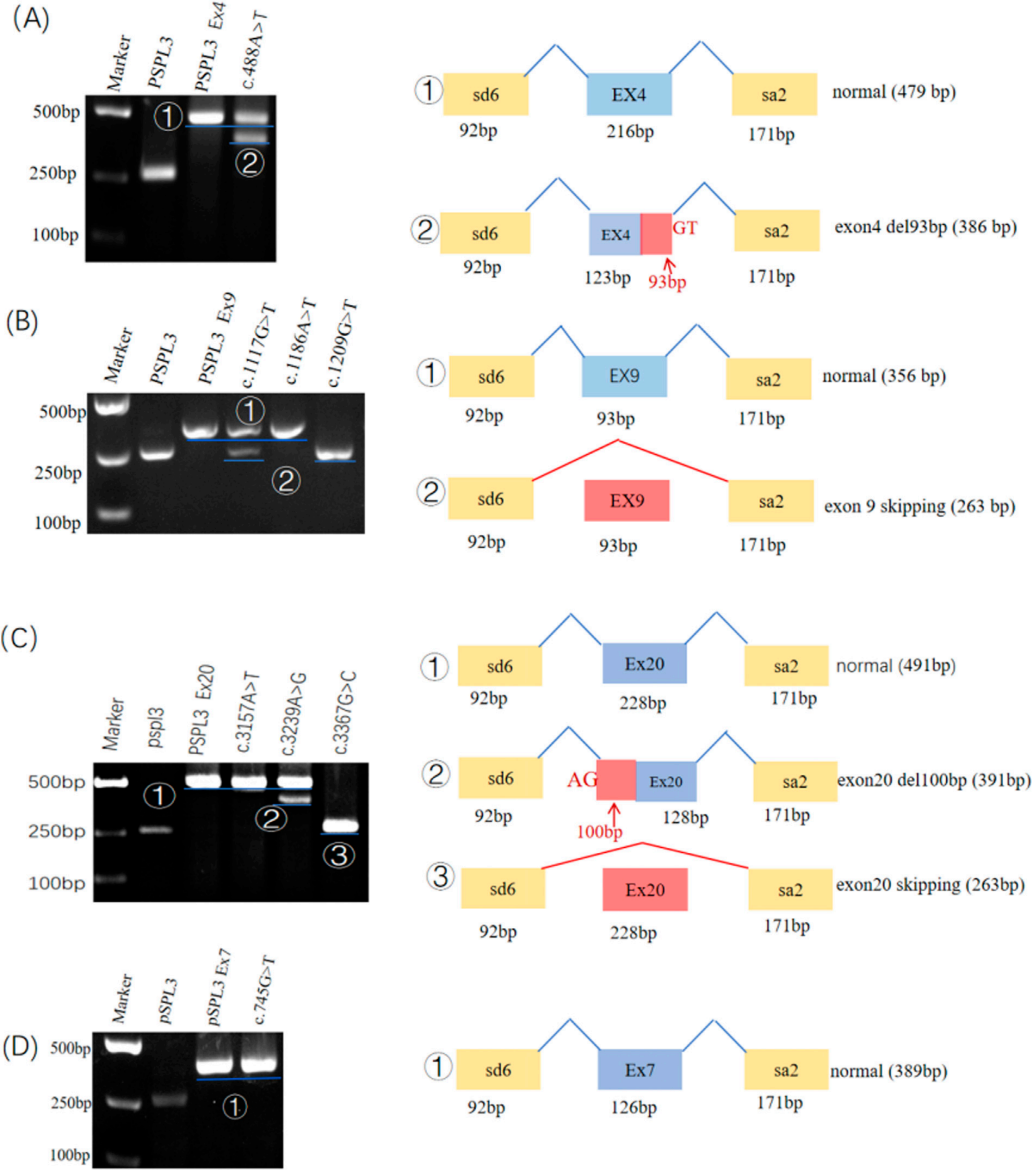
Electrophoresis results and transcriptional analysis of wild-type and mutant minigene constructs: the electrophoresis results are presented on the left, while splicing events and transcripts are depicted on the right. **(A)** The wild type showed a single normal band. Variants c.488A>T showed both normal and abnormal (93 bp deletion) bands. **(B)** The wild type and c.1186A>T showed normal splicing. Variant c.1117G>T exhibited both normal and abnormal (exon 9 skipping) band. Variants c.1209G>T caused corresponding exon 9 skipping. **(C)** The wild type and c.3157A>T showed normal splicing. Variants c.3239A>G showed both normal and abnormal (100 bp deletion) bands. Variants c.3367G>C caused corresponding exon skipping. **(D)** The wild type and variant c.745G>T showed normal splicing.

A total of all missense and nonsense variants compiled in the CFTR database was analyzed using the bioinformatics software. All screened variants were analyzed with BDGP and SpliceAI for splice site prediction and with HSF for ESE/ESS estimation algorithms *in silico*. The enrolled missense variants were as follows: c.454A>G (p. Met152Val),c.488A>T (p. Lys163Met) in exon 4, c.745G>T (p. Asp249Tyr) in exon 7, c.1117G>T (p. Asp373Tyr), c.1186A>T (p. Asn396Tyr), c.1209G>C (p. Glu403Asp) and c.1209G>T (p. Glu403Asp) in exon 9, c.3157A>T (p. Thr1053Ser), c.3239A>G (p. Gly1123Arg), and c.3367G>C (p. Gly1123Arg) in exon 20. The missense variant c.454A>G (p.Met152Val) (Lee et al., 2017) and variant c.1209G>C (p.Glu403Asp) (Bergougnoux et al., 2023) have been reported to alter pre-mRNA splicing, so these two single nucleotide substitutions were excluded. The predictions of eight candidate variants were presented in Table 1. Different control minigenes were constructed, including CFTR WT sequences of exon 4 (pSPL3-CFTR Ex4), exon7 (pSPL3-CFTREx7), exon 9 (pSPL3-CFTR Ex9), and exon 20 (pSPL3-CFTR Ex20). Mutant minigenes were generated by site-directed mutagenesis. Finally, five exon variants were shown to result in aberrant splicing *in vitro* (Figure 2, Table 2).

**TABLE 1:** Bioinformatics analysis of exonic variants screened in this study.

Variant	Amino acid	Exon (length)	Position/AG/GT	BDGP	SpliceAI	HSF
c.488A>T	p. Lys163Met	4 (216 bp)	2 bp from 5′-GT^a^	DS: 0.75→0.64^b^	DL 0.74^c^	−3^d^
c.745G>T	p. Asp249Tyr	7 (126 bp)	2 bp from 3′-AG^a^	AS:0.22→0.52^b^	NA	−6^d^
c.1117G>T	p. Asp373Tyr	9 (93 bp)	1 bp from 3′-AG^a^	AS:0.57→0.69^b^	AL 0.23^c^	−5^d^
c.1186A>T	p. Asn396Tyr	9 (93 bp)	24 bp from 5′-GT^a^	DS: 0.02→0.15^b^	DG 0.44^c^	−6^d^
c.1209G>T	p. Glu403Asp	9 (93 bp)	1 bp from 5′-GT^a^	DS: 0.14→0^b^	DL 0.89^c^	−4^d^
c.3157A>T	p. Thr1053Ser	20 (228 bp)	18 bp from 3′-AG^a^	AS: 0.15→0.23^b^	AG 0.54^c^	−4^d^
c.3239A>G	p. Lys1080Arg	20 (228 bp)	100 bp from3′-AG^a^	AS: 0.03→0.74^b^	AG 0.45^c^	New AS
c.3367G>C	p. Gly1123Arg	20 (228 bp)	1 bp from 5′-GT^a^	DS: 0.91→0.29^b^	DL 0.98^c^	broken WT DS

Abbreviations: AS, acceptor splice site; DS, donor splice site; ESE, exonic splicing enhancer; ESS, exonic splicing silencer; NA, not applicable; WT, wild type.

^a^ Position relative to the nearest 3′-AG/5′-GT dinucleotides.

^b^ Alterations in the BDGP scores.

^c^ AG, Acceptor Gain, AL: Acceptor Loss, DG: Donor Gain, DL: Donor Loss. The score is used to predict the possibility of variation affecting splicing in the current reading frame. The recommended threshold is > 0.5, but 0.2 < score <0.5 may also affect splicing, and >0.8 is very likely to affect splicing.

^d^ Values −3, −4, −5, and −6 indicate ESE/ESS motif ratio.

**TABLE 2:** Splicing outcomes for exonic variants with impact on splicing.

Variant	Amino acid	Affected motifs	Aberrant transcripts	Protein effect
c.488A>T	p. Lys163Met	Canonical splice site and ESEs/ESSs	Partial deletion (93 bp) of exon4 r.397_489del;	p. (Val133_Lys163del) 31 amino acids loss in TMD1
c.1117G>T	p. Asp373Tyr	Canonical splice site and ESEs/ESSs	Skipping of exon9 (93 bp) r.1117_1209del;	p. (Asp373_Glu403del) 31 amino acids loss in TMD1
c.1209G>T	p. Glu403Asp	Canonical splice site and ESEs/ESSs	Skipping of exon 9 (93 bp) r.1117_1209del;	p. (Asp373_Glu403del) 31 amino acids loss in NBD1
c.3239A>G	p. Lys1080Arg	Canonical splice site	Partial deletion (100 bp) of exon20 r.3140_3239del;	p. (Gly1047Glufs*3) truncated protein in TMD2
c.3367G>C	p. Gly1123Arg	Canonical splice site	Skipping of exon 20 (228bp) r.3140_3367del;	p. (Gly1047_Thr1122del) 76 amino acids loss in TMD2

### 3.1 Variants c.488A>T (p. Lys163Met) induced truncated exon 4

Variant c.488A>T (p. Lys163Met), located at the 2nd nucleotide position from the 3′end of exon 4 (216 bp), was predicted to generate a cryptic splice site located at the first 124 bp from the 5′end of exon 4 (score: 0.12; ctttattGTgaggac) by BDGP. Using HSF 3.1 software for *in silico* analysis revealed that this variant not only disrupts three ESEs but also creates two ESSs. Similarly, Splice AI indicated that it may cause exon 4 to lose the classical donor site (score: 0.74). The minigene assay results identified that the WT and mutant minigenes generated different cDNA products, respectively. The WT Lane just demonstrated one fragment of 479 bp that contains exon 4, SD, and SA of the pSPL3, whereas the mutant minigene c.488A>T produced two fragments with lengths of 479 bp and 386 bp, respectively (Figure 2A), which correspond to the normal transcripts carrying the variant c.488A>T and the abnormal ones with the missing of the last 93 bp of exon 4, confirmed by sequencing.

### 3.2 Variants c.1117G>T (p. Asp373Tyr), c.1209G>T (p. Glu403Asp) all resulted in skipping of exon 9

Variant c.1117G>T (p. Asp373Tyr), located at the first nucleotide position from the 5′end of exon 9, reduced the score of the 3′ss from 0.57 to 0.07 according to *in silico* analysis using BDGP. In silico analysis by HSF 3.1 software demonstrated that this variant not only disrupts one ESE but also creates five ESSs. By sequencing analysis of c.1117G>T (p. Asp373Tyr) for all bands, it was confirmed that the larger fragment (356 bp) corresponds to correctly spliced exons (CFTR exon 9 and flanked by exon SD and exon SA of the pSPL3 vector) and the smaller fragment (263 bp) corresponds to a transcript without exon 9 (Figure 2B).

Variant c.1209G>T (p. Glu403Asp), located at the first nucleotide position from the 3′end of exon 9, reduced the score of the 5′ss from 0.14 to 0 based on *in silico* analysis using BDGP. In silico analysis by HSF 3.1 software demonstrated that this variant not only disrupts six ESEs but also creates one ESS. Splice AI indicated that it may cause exon 9 to lose the classical donor site (score: 0.89). The RT-PCR results showed that there was one band for the mutant minigene. By sequencing analysis of this band, the mutant lane of c.1209G>T identified one major aberrant transcript of 263 bp, indicating a complete skipping of exon 9 (Figure 2B).

### 3.3 Variants c.3239A>G (p.Lys1080Arg) induced truncated exon 20,c.3367G>C (p.Gly1123Arg) resulted in skipping of exon 20

Variants c.3239A>G (p. Lys1080Arg), located in the middle of exon 20, was predicted to generate a cryptic splice site located at the first 101 bp from the 5′end of exon 20 (score: 0.03; tgt​tcc​aca​aAG​ctc​tga​att​ac) by BDGP. Similarly, *in silico* analysis by HSF 3.1 software demonstrated that variant c.3239A>G activated a cryptic acceptor site. The minigene assay result identified that the WT and mutant minigenes generated different cDNA products, respectively. The WT lane just demonstrated one fragment of 491 bp that contains exon 20, SD, and SA of the pSPL3, whereas the mutant minigene c.3239A>G produced two fragments with lengths of 491 bp and 391 bp, respectively. One of the bands with a size of 491 bp corresponded to the correctly spliced exon 20, while another product (391 bp) was alternatively spliced (Figure 2C), resulting in a deletion of the first 100 bp of exon 20.

Variant c.3367G>C (p. Gly1123Arg), located at the first nucleotide position from the 3′end of exon 20, reduced the score of the 5′ss from 0.91 to 0.29 based on *in silico* analysis using BDGP. SpliceAI indicated that it may lose the donor site (score: 0.98). The RT-PCR results showed that there was one band for the mutant minigene (Figure 2C). By sequencing analysis of this band, it was confirmed that the fragment corresponds to a transcript without exon 20. The mutant lane of c.3367G>C identified one major aberrant transcript of 263 bp, indicating a complete skipping of exon 20 (Supplementary Figure S2C).

### 3.4 Variant c.745G>T (p. Asp249Tyr), c.1186A>T (p. Asn396Tyr) and c.3157A>T (p. Thr1053Ser) did not alter the pre-mRNA splicing

Variant c.745G>T (p. Asp249Tyr), located at the 2nd nucleotide position from the 5′end of exon 7, was postulated to be important for correct splicing and might lead to abnormal splicing. In silico analysis by HSF 3.1 software demonstrated that this variant not only disrupts seven ESEs but also creates two ESSs. Variant c.1186A>T (p. Asn396Tyr) and c.3157A>T (p. Thr1053Ser) were also postulated to be important for correct splicing and might lead to abnormal splicing, despite their location away from the canonical splice site. However, the results of the minigene assays showed that they have no influence on the splicing of pre-mRNA (Figure 2).

## 4 Discussion

Alteration of the pre-mRNA splicing has long been considered a potential cause of rare genetic diseases, with significant implications for our understanding of conditions such as cystic fibrosis (CF). Previous studies have found that variants can affect splicing by causing exon skipping or by activating new cryptic splice sites (Cartegni et al., 2002). Around 7% of reported CFTR variants are classified as splicing variants, which is likely an underestimate. In addition, the current study of the CFTR gene focuses on the protein structure and function exploration, which are relatively mature, but the pre-mRNA splicing studies are lacking. It is therefore crucial to incorporate RNA-level analyzes into routine practice in genetic disease research, particularly when investigating splicing defects associated with exonic variants (Joynt et al., 2020). The most appropriate method for identifying splicing aberrations is to study the patient RNA. However, the instability of transcripts in tissues often poses challenges in obtaining adequate samples. When patient samples cannot be collected, minigene-based technologies have emerged as reliable and straightforward alternatives (Martin et al., 2021). In this study, we constructed pSPL3 minigenes and transfected them into HEK293T and BEAS-2B cells to examine their mutational effect of previously described CFTR missense or nonsense variants. Ultimately, our results demonstrated that five candidate variants lead to alternative splicing in precursor RNA. The Ideozu manuscript (Ideozu et al., 2024) shows that many variants are not associated with clinical outcomes. Nonetheless, these typically silent variants can affect the severity of clinically relevant variants (Kosova et al., 2010). Thus, the field is beginning to appreciate that the broader context of the gene can affect the impact of a clinically relevant variant.

It is reported that the sequencing results of human 5ʹss sequence (CAG/GUAAGU) show that there are more than 9000 nucleotide sequence variations in the −3 to +5 region of GU dinucleotide, and the sequence elements around the splicing site are very short and poorly conserved. This makes 5ʹ splice site recognition and selection plastic (Hicks et al., 2010; Roca et al., 2012). Variants, near to classical splice sites, may alter the strength of the splice site, thereby causing the corresponding exons to jump. In our study, variant c.3367G>C is located at the last nucleotide of exon 20. As predicted (score 0.91→0.29, as assessed by BDGP, Table 1; Figure 1), this variant weakens the strength of the splice sites, leading to the skipping of exon 20. Analysis from the predicted protein level, skipping of exon 20 results in in-frame deletions (codon 3140-3367), leading to a loss of 76 amino acids. The predicted protein product of this splice variant would lack 76 amino acids that is likely to affect protein synthesis, processing, trafficking and/or function.

Similarly, exon 9 exhibited a weak 5ʹ splice donor site (score 0.14, as assessed by BDGP, Table 1; Figure 1),making it more susceptible to changes in splicing. Variant c.1209G>T is located at the last nucleotide of the exon 9, reduced the score of the 5ʹss from 0.14 to 0 based on *in silico* analysis using BDGP. In addition, the reduction of the proportion of ESEs/ESSs may account for this result to some extent. Minigene analysis demonstrated that this alteration could result in skipping of exon 9. Variant c.1209G>T caused in-frame deletion of 31 amino acids, located within the NBD1 domain. The NBD1 domain plays a critical role in the core functionality of the CFTR protein, as it binds ATP and maintains the functional stability of CFTR (Hwang et al., 2023). Exon 9 encodes the first 21% of the NBD1 domain. The defective protein encoded by the missing exon 9 transcript is misfolded and cannot be transferred from the endoplasmic reticulum to the Golgi complex and into the cell membrane. As a result, the normal CFTR Cl-channels in the cell membrane are significantly reduced and can lead to a disease-causing cystic fibrosis phenotype (Delaney et al., 1993; Pagani et al., 2000).

Variants at the 3′ splicing site can also influence splicing efficiency. The 3′ splice site region contains multiple conserved elements, including the branch point, poly-pyrimidine tract, and the AG dinucleotide at the 3′ end of the intron. The mechanism of the 3′ splice site may be more complex. The c.1117G>T variant, located at the first nucleotide of exon 9, produces both a correctly spliced transcript and an abnormal transcript in which exon 9 is completely skipped. The c.1117G>T variant may have dual effects: on the one hand, some CFTR transcripts are detrimental due to exon 9 skipping, resulting in the deletion of the corresponding amino acid sequence in transmembrane domain 1 (TMD1); On the other hand, the remaining mRNA contains a single amino acid substitution, which may lead to dysfunction of chloride channel proteins. Notably, the c.1117G>T variant yields only a limited number of aberrant transcripts. This simultaneous production of correctly spliced and aberrant transcripts allows for relative proportion analysis, contributing to the overall assessment of pathogenicity (Bergougnoux et al., 2023). CFTR variants lacking exon 9 were reported soon after the identification and cloning of CFTR with these transcripts failing to mature and function (Chu et al., 1991; Radpour et al., 2008). A subsequent report from the same laboratory (Chu et al., 1991) reported that the shorter poly-T track at the splicing/branch site of exon 9 in the CFTR gene, the higher the proportion of in-frame skipping of exon 9 in CFTR mRNA in respiratory epithelial cells. Although this research elucidates the genetic basis for the extent of exon 9 skipping, the molecular mechanisms controlling this process remain unclear. The splicing effect of 1117G>T in human tissues must be determined.

Different from c.1117G>T variant, the c.488A>T and c.3239A>G variants activated hidden splice sites, leading to the production of both normal transcripts and abnormal transcripts with partial deletions. For instance, Variant c.488A>T is located at the 2nd nucleotide position from the 3′end of exon 4. Variant c.488A>T activates a cryptic splice donor site (93 bp from the 3′ end of exon 4)that competes with the natural splice site. This novel splice site is favored over the natural one, resulting in abnormal splicing characterized by the missing of the last 93 bp of exon 4 (Supplementary Figure S2; Figure 2A). In this study, *in vitro* minigene analysis revealed that it not only generated a transcript containing c.488A>T but also produced a transcript with partial deletion of exon 4 (93bp), resulting in protein changes in the important domain TMD1 (in-frame deletion 31 amino acids). Analysis from the predicted protein level, variant c.488A>T caused in-frame deletion of amino acids, located within TMD1 domains. The pathogenicity of the two transcripts caused by this variant is difficult to predict. Similarly, variant c.3239A>G is located in the middle of exon 20 and activates a cryptic acceptor site (101 bp from the 5′end of exon 20)that competes with the natural one. Minigene assays demonstrated that this variant generates both transcript containing c.3239A>G and abnormal transcripts that are missing the first 100 bp of exon 20 (Figure 2C). Analysis from the predicted protein level, the partial deletion of exon 20 alters the open reading frame, leading to the substitution of glutamic acid for glycine at codon 1047, followed by a frameshift at this position and premature truncation after the addition of two amino acids (p. Gly1047Glufs*3). The aberrantly spliced transcripts result in the production of truncated protein in the TMD2 domain. Variant c.3239A>G produced only a limited number of aberrant transcripts. Additionally, it has also been reported that some splicing variants generate abnormal transcripts harboring an in-frame premature termination codon (PTC), which are eliminated by the mRNA surveillance mechanism, thereby reducing the accumulation of toxic truncated proteins (Supek et al., 2021). Collectively, these results confirm that the c.488A>T and c.3239A>G variants activate cryptic splice sites within exons and may result in the partial deletion of crucial structural domains of the CFTR protein. Therefore, we recommend considering the activation of cryptic splice sites when evaluating the pathogenicity of exonic and intronic variants (Lee et al., 2017).

The true splicing effect of these variants needs to be confirmed by analyzing the mRNA from the patient, which we did not have yet. At the same time, our experimental method has certain limitations, as low-expression bands are sometimes difficult to detect on the gel. Therefore, it is appropriate to use high-sensitivity fluorescent probes in subsequent studies to improve detection capability and optimize the experimental results. Moreover, it is important to note that minigenes only evaluate the impact of single point variants and cannot fully simulate conditions within the human body, as variants can influence each other. Linking the fundamental defects caused by variants to their therapeutic implications has advanced the research and development of cystic fibrosis medications (Molinski et al., 2014). In addition, it was reported that the variant c.2908G>C (predicted effect G970R) was shown to respond well to ivacaftor (potentiator VX‐770) *in vitro*, whereas individuals with cystic fibrosis carrying this variant that were entered into a clinical trial did not respond. And studies have confirmed that c.2908G>C induced 78.5% ± 3.2 of CFTR transcript skipping exon 17% and 21.5% ± 3.2 of transcript harboring the 177-nucleotide deletion. Therefore, aberrant splicing events may explain why some exonic variants cause a lack of responsiveness to drugs (Joynt et al., 2020).

Variant c.745G>T (p. Asp249Tyr), c.1186A>T (p. Asn396Tyr) and c.3157A>T (p. Thr1053Ser) were predicted to alter the splicing process by influencing ESEs and ESS motifs or changing the recognition of classic splice sites. However, results from our minigene assays indicated that these variants did not influence pre-mRNA splicing, which is inconsistent with the predictions made by software. This discrepancy highlights certain limitations in software predictions, particularly their inability to account for all underlying splicing patterns (Xin et al., 2022). Unfortunately, in the present study, we were unable to obtain patient RNA for comparative analysis. Furthermore, we did not assess the expression of wild-type and mutant proteins in cells nor evaluate their function *in vitro*. For instance, the variant c.3700 A>G leads to CF disease due to abnormal splicing rather than the predicted missense variant (Molinski et al., 2014). Therefore, while computational predictions have their limitations, they should be complemented with *in vitro* experimental validation. Expression minigenes (EMGs) offer a powerful experimental approach for the clinical interpretation of splicing site variations and the improvement of computational tools (Sharma et al., 2014). Interestingly, the E831X variant, initially thought to be a nonsense variant, actually affected acceptor sites and removed new PTC, leading to the mild to asymptomatic phenotype (Hinzpeter et al., 2010). Overall, the mRNA splicing effects warrant further investigation. Therefore, more research will be needed in the future to validate these findings.

Currently, antisense oligonucleotides (ASOs) and small molecules demonstrate promising results in splicing-directed therapy for rare genetic diseases. ASOs that target splice sites or cis-acting elements can induce either exon skipping or inclusion while sterically blocking the interaction of the splicing machinery (Love et al., 2023). This ASO strategy presents a promising therapeutic approach for the specific correction of alternative splicing. Similarly, gene editing technologies hold clinical potential to treat various single-gene genetic diseases, offering hope for curing a range of rare genetic disorders at the genetic level.

## 5 Conclusion

In conclusion, we have carried out an analysis of exonic variants in CFTR related to CF through bioinformatics predictions and minigene assays. Five variants (c.488A>T, c.1117G>T, c.1209G>T, c.3239A>G,c.3367G>C) were confirmed to affect pre-mRNA splicing. This study highlights the important role of assessing the effects of single nucleotide variants at the mRNA level, as well as the effectiveness of minigene splicing analysis. This approach may enhance our understanding of the pathogenesis of CF and contribute to optimizing therapeutic strategies.

## Data Availability

The datasets presented in this study can be found in online repositories. The names of the repository/repositories and accession number(s) can be found in the article/Supplementary Material.

## Abbreviations

CFTR, Cystic fibrosis transmembrane conductance regulator; BDGP, Berkeley *Drosophila* Genome Project; HEK 293T, Human epithelial kidney 293T; BEAS-2B, Bronchial Epithelium transformed with Ad12-SV40 2B.
