# Supplementary material for: Identified five variants in CFTR gene that alter RNA splicing by minigene assay
Source: Front Genet. 2025 Mar 20;16:1543623. doi: 10.3389/fgene.2025.1543623 (PMC11965618; doi:10.3389/fgene.2025.1543623)
Supplement: Supplementary file 1 [file Table1.docx]

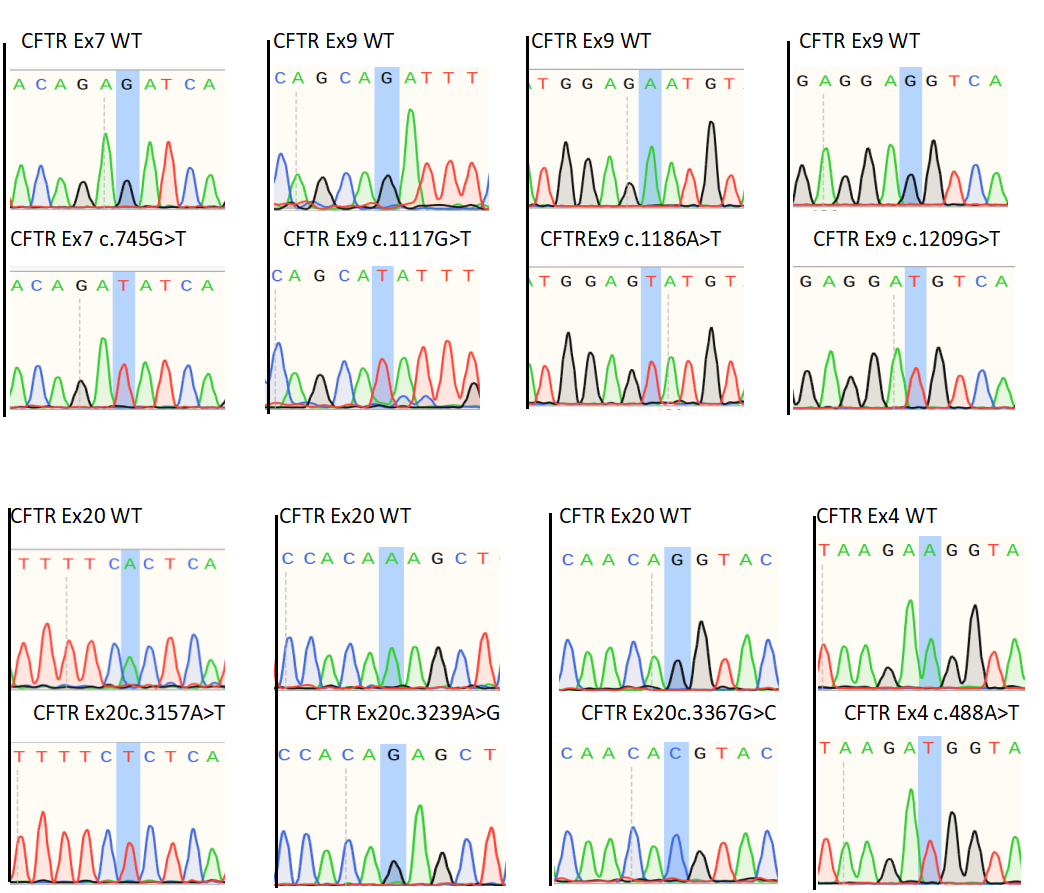


**Supplementary Figure 1.** The sanger sequencing results of all constructed recombinant plasmids. The blue boxes indicate the mutation sites.
