# Supplementary material for: Identified five variants in CFTR gene that alter RNA splicing by minigene assay
Source: Front Genet. 2025 Mar 20;16:1543623. doi: 10.3389/fgene.2025.1543623 (PMC11965618; doi:10.3389/fgene.2025.1543623)
Supplement: Supplementary file 2 [file Table7.docx]

Supplementary Table 4 spliceAI scores and their relative location for each variant studied

| variant | [spliceAI](https://github.com/Illumina/SpliceAI) AG | [spliceAI](https://github.com/Illumina/SpliceAI) AL | [spliceAI](https://github.com/Illumina/SpliceAI) DG | [spliceAI](https://github.com/Illumina/SpliceAI) DL |
| --- | --- | --- | --- | --- |
| c.488A>T | 0(-14) | 0(2) | 0(41) | 0.74(1) |
| c.745G>T | 0(-1) | 0.03（1） | 0（32） | 0（1） |
| c.1117G>T | 0（15） | 0.23（0） | 0（0） | 0（13） |
| c.1186A>T | 0(23) | 0(-50) | 0.44(-2) | 0.03(23) |
| c.1209G>T | 0(20) | 0(0) | 0.02(-21) | 0.89(0) |
| c.3157A>T | 0.54(17) | 0.06(-13) | 0(17) | 0(-13) |
| c.3239A>G | 0.45(1) | 0(3) | 0(1) | 0(3) |
| c.3367G>C | 0(36) | 0(1) | 0(-28) | 0.98(0) |

AG:Acceptor Gain, AL:Acceptor Loss, DG:Donor Gain, DL:Donor Loss.

Δ score 0-1 (relative position in bp), thresholds ≥ 0.2|0.5|0.8 for impact.
