# Supplementary material for: Identified five variants in CFTR gene that alter RNA splicing by minigene assay
Source: Front Genet. 2025 Mar 20;16:1543623. doi: 10.3389/fgene.2025.1543623 (PMC11965618; doi:10.3389/fgene.2025.1543623)
Supplement: Supplementary file 3 [file Table2.docx]

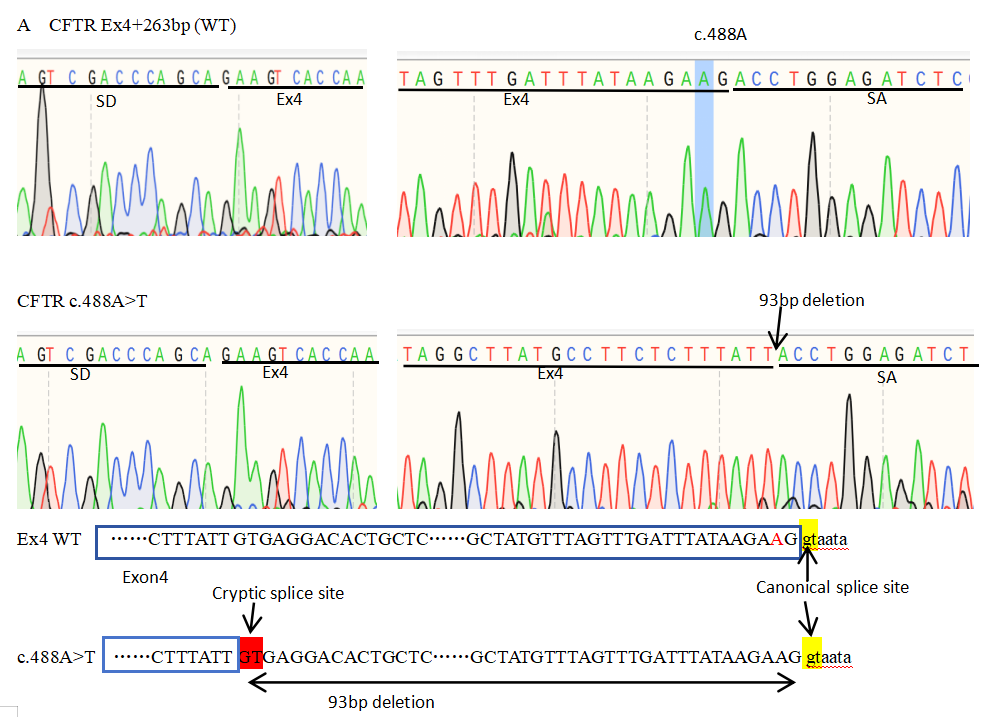


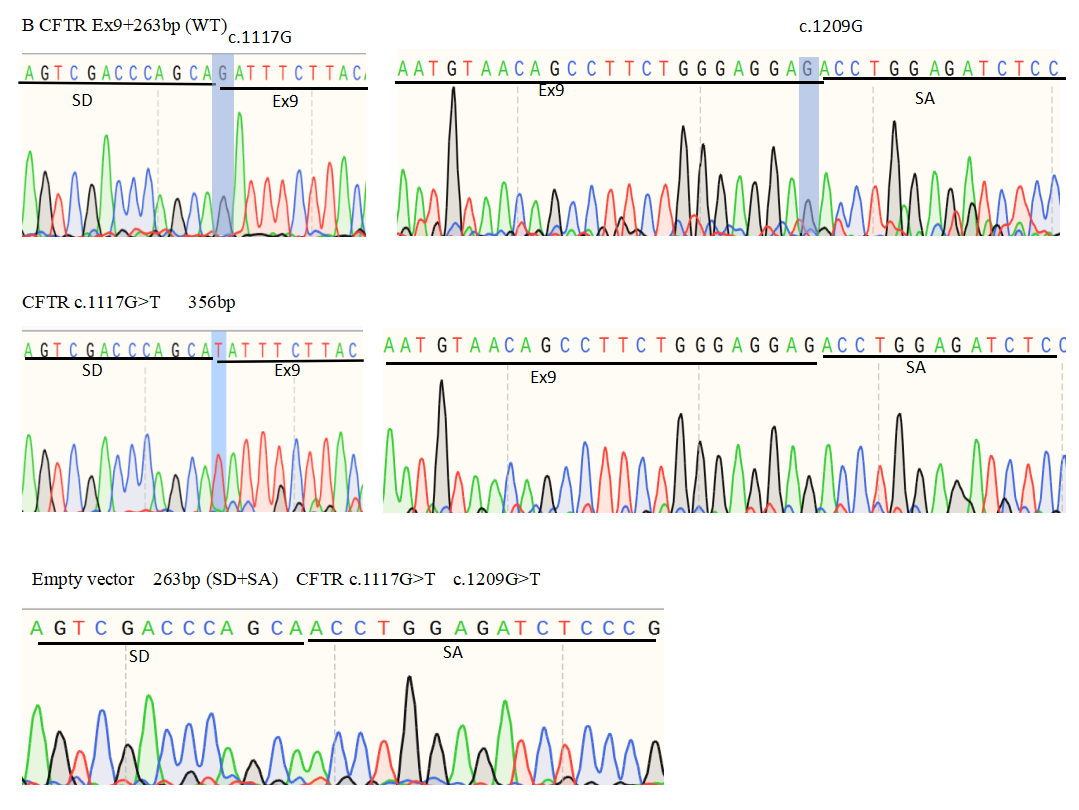


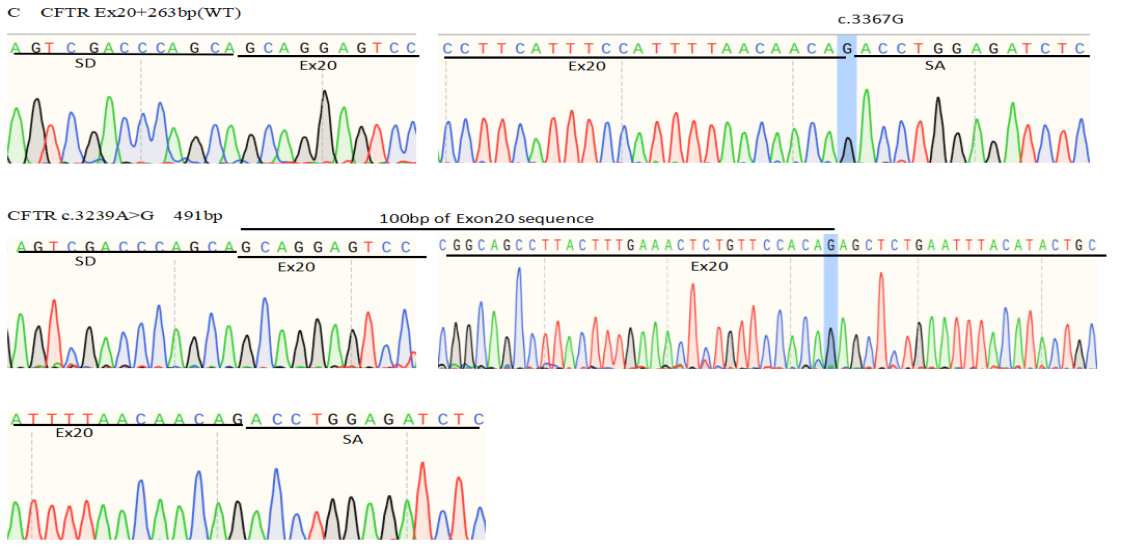


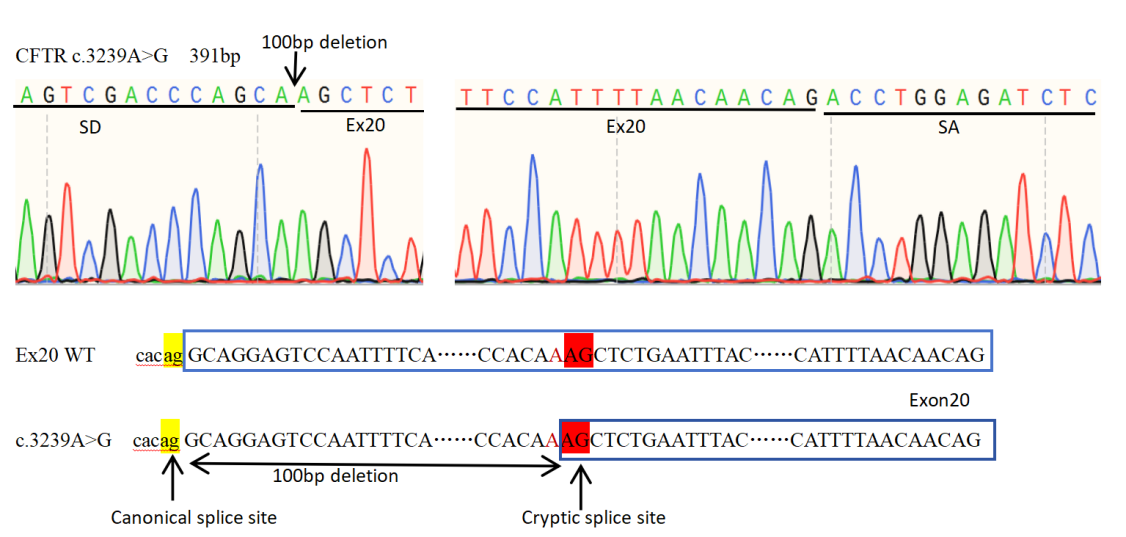


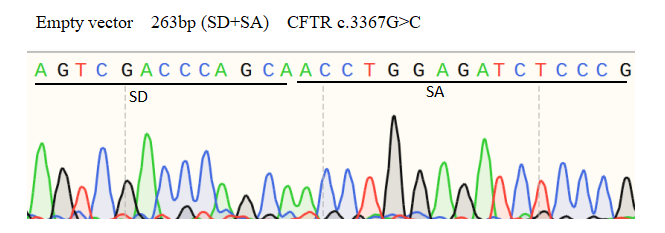


**Supplementary Figure 2.** A:Sequencing results of aberrant transcripts for variants in exon 4.Cryptic splicing identified in variants c.488A>T. Sequencing analysis below confirms cryptic splicing of variant c.488A>T, resulting in a 93 bp deletion at the 3' end of exon4 (upper panel). The base marked in red is the c.488A>T mutation. Schematic representation of canonical/cryptic splice sites shows that variant c.488A>T uses an exonic cryptic donor site (lower panel). Canonical/cryptic splice sites are lined out.

B:Sequencing results of aberrant transcripts for variants in exon 9 .The sequencing results were continuous, but the presentation was disconnected due to space limitations.

C:Sequencing results of aberrant transcripts for variants in exon 20.Cryptic splicing identified in variants c.3239A>G. Sequencing analysis below confirms cryptic splicing of variant c.3239A>G, resulting in a 100 bp deletion at the 5' end of exon20 (upper panel). The base marked in red is the c.3239A>G mutation. Schematic representation of canonical/cryptic splice sites shows that variant c.3239A>G uses an exonic cryptic donor site (lower panel). Canonical/cryptic splice sites are lined out.
