# Supplementary material for: Identified five variants in CFTR gene that alter RNA splicing by minigene assay
Source: Front Genet. 2025 Mar 20;16:1543623. doi: 10.3389/fgene.2025.1543623 (PMC11965618; doi:10.3389/fgene.2025.1543623)
Supplement: Supplementary file 4 [file Table5.docx]

Supplementary Table 2 Primer sequences for introducing mutations into exons.

| Mutations | sequences (5'-3') |
| --- | --- |
| CFTR EXON4-488-PSPL3-F | GATTTATAAGATGGTAATACTTCC |
| CFTR EXON4-488-PSPL3-R | GGAAGTATTACCATCTTATAAATC |
| CFTR EXON7-745-PSPL3-F | GATTTACAGATATCAGAGAGC |
| CFTR EXON7-745-PSPL3-R | GCTCTCTGATATCTGTAAATC |
| CFTR EXON9-1117-PSPL3-F | GCTCTCTTTTATAAATAGTATTTC |
| CFTR EXON9-1117-PSPL3-R | GAAATACTATTTATAAAAGAGAGC |
| CFTR EXON9-1186-PSPL3-F | GTGATGGAGTATGTAACAGC |
| CFTR EXON9-1186-PSPL3-R | GCTGTTACATACTCCATCAC |
| CFTR EXON9-1209-PSPL3-F | CTGGGAGGATGTCAGAAT |
| CFTR EXON9-1209-PSPL3-R | ATTCTGACATCCTCCCAG |
| CFTR EXON20-3157-PSPL3-F | GGAGTCCAATTTTCTCTCATCTTG |
| CFTR EXON20-3157-PSPL3-R | CAAGATGAGAGAAAATTGGACTCC |
| CFTR EXON20-3239-PSPL3-F | CTCTGTTCCACAGAGCTCTGAATT |
| CFTR EXON20-3239-PSPL3-R | AATTCAGAGCTCTGTGGAACAGAG |
| CFTR EXON20-3367-PSPL3-F | CATTTTAACAACACGTACTATGAACTC |
| CFTR EXON20-3367-PSPL3-R | GAGTTCATAGTACGTGTTGTTAAAATG |
