# Supplementary material for: Identified five variants in CFTR gene that alter RNA splicing by minigene assay
Source: Front Genet. 2025 Mar 20;16:1543623. doi: 10.3389/fgene.2025.1543623 (PMC11965618; doi:10.3389/fgene.2025.1543623)
Supplement: Supplementary file 5 [file Table3.docx]

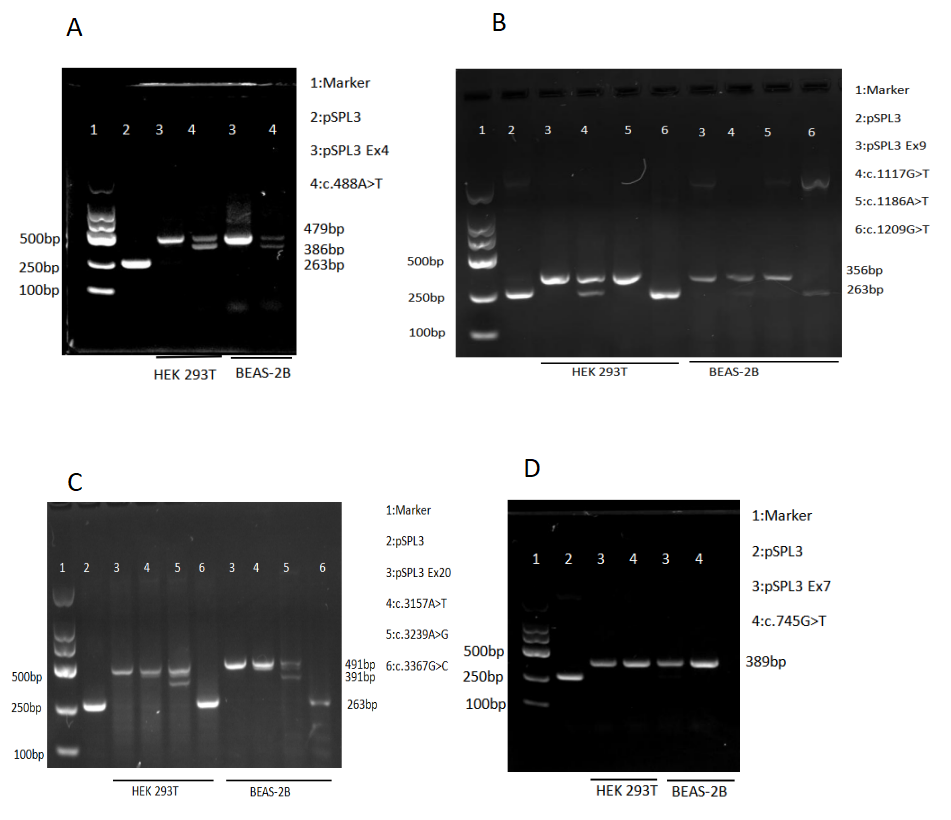


**Supplementary Figure 3.** The full-size gels for minigene assays.

The legend for each lane is shown in the upper right of each image. The size of the empty vector pSPL3 is 263 bp.

**(**A**)** The full-size gel of variants in exon 4. **(**B**)** The full-size gel of variant in exon 9. (C**)** The full-size gel of variants in exon 20.

(D)The full-size gel of variants in exon 7.
