# Supplementary material for: Identified five variants in CFTR gene that alter RNA splicing by minigene assay
Source: Front Genet. 2025 Mar 20;16:1543623. doi: 10.3389/fgene.2025.1543623 (PMC11965618; doi:10.3389/fgene.2025.1543623)
Supplement: Supplementary file 6 [file Table4.docx]

Supplementary Table 1 Primer sequences for amplifying exons

| Exons | sequences (5'-3') |
| --- | --- |
| CFTR EXON4-PSPL3-F | CCG CTCGAG ACTTGTCTCCCACTGTTGCT |
| CFTR EXON4-PSPL3-R | CTA GCTAGC ACAACAGAGGCAGTTTACAG |
| CFTR EXON7-PSPL3-F | CCG CTCGAG TTAGTCAAGCCACTTCACCT |
| CFTR EXON7-PSPL3-R | CTA GCTAGC CTACAGCCCATGAAAGTGAA |
| CFTR EXON9-PSPL3-F | CCG CTCGAG CAAGGAAGAATCAGTTGTAT |
| CFTR EXON9-PSPL3-R | CTA GCTAGC ATCCTCCTTCCAGTTCTACCAG |
| CFTR EXON20-PSPL3-F | CCG CTCGAG TATTCAAAGAATGGCACCAG |
| CFTR EXON20-PSPL3-R | CTA GCTAGC TGTGAAAACAGGGATAATAC |
