# Supplementary material for: Identified five variants in CFTR gene that alter RNA splicing by minigene assay
Source: Front Genet. 2025 Mar 20;16:1543623. doi: 10.3389/fgene.2025.1543623 (PMC11965618; doi:10.3389/fgene.2025.1543623)
Supplement: Supplementary file 7 [file Table6.docx]

Supplementary Table 3

| variant | Amino acid | ClinVar  Classification（N） | dbSNP | References | gnom AD  Allele Frequency |
| --- | --- | --- | --- | --- | --- |
| c.488A>T | p. Lys163Met | Conflicting classifications of pathogenicity (2) | rs1562889435 | NA | NA |
| c.745G>T | p. Asp249Tyr | uncertain significance (1) | rs1798959414 | Prach L et al. (2013)^(1)^ | 6.319e-7 |
| c.1117G>T | p. Asp373Tyr | uncertain significance (1) | NA | NA | 6.926e-7 |
| c.1186A>T | p. Asn396Tyr | uncertain significance (3) | rs753143757 | NA | 1.88e-6 |
| c.1209G>T | p. Glu403Asp | likely pathogenic (2) | NA | NA | NA |
| c.3157A>T | p. Thr1053Ser | uncertain significance (2) | rs1792386620 | NA | 4.35e-6 |
| c.3239A>G | p. Lys1080Arg | uncertain significance (2) | rs564165440 | NA | 2.04e-5 |
| c.3367G>C | p. Gly1123Arg | not provided (1) | rs397508546 | Hughes DJ et al. (1996)^(2)^ | 6.249e-7 |

ClinVar last accessed March 2024. In brackets, N submitters supporting each classification. NA, not applicable.

1. Prach L, Koepke R, Kharrazi M, Keiles S, Salinas DB, Reyes MC, et al. Novel CFTR variants identified during the first 3 years of cystic fibrosis newborn screening in California. J Mol Diagn. 2013;15(5):710-22.DOI:10.1016/j.jmoldx.2013.05.006.

2. Hughes DJ, Hill AJ, Macek M, Jr., Redmond AO, Nevin NC, Graham CA. Mutation characterization of CFTR gene in 206 Northern Irish CF families: thirty mutations, including two novel, account for approximately 94% of CF chromosomes. Hum Mutat. 1996;8(4):340-7.DOI:10.1002/(sici)1098-1004(1996)8:4<340::Aid-humu7>3.0.Co;2-b.
